# Immigration as pathogenic: a systematic review of the health of immigrants to Canada

**DOI:** 10.1186/1475-9276-9-27

**Published:** 2010-11-24

**Authors:** Fernando G De Maio

**Affiliations:** 1 Department of Sociology and Anthropology, Simon Fraser University, Burnaby, British Columbia, Canada V5A 1S6

## Abstract

This review investigates the health of immigrants to Canada by critically examining differences in health status between immigrants and the native-born population and by tracing how the health of immigrants changes after settling in the country. Fifty-one published empirical studies met the inclusion criteria for this review. The analysis focuses on four inter-related questions: (1) Which health conditions show transition effects and which do not? (2) Do health transitions vary by ethnicity/racialized identity? (3) How are health transitions influenced by socioeconomic status? and (4) How do compositional and contextual factors interact to affect the health of immigrants? Theoretical and methodological challenges facing this area of research are discussed and future directions are identified. This area of research has the potential to develop into a complex, nuanced, and useful account of the social determinants of health as experienced by different groups in different places.

## Introduction

Patterns of immigrant health have received considerable attention in the social science and medical literature in the past two decades, particularly in the United States [1-3], where researchers have focused on the 'paradox' of good health given relatively poor socioeconomic conditions among Hispanic immigrants. In Canada, research on the health of immigrants has engaged with contrasting notions of 'sick immigrants' and 'healthy immigrants' [4]; the former describing immigrants as carriers of disease and as burdens on health and social welfare systems, and the latter acknowledging that because of a number of factors - including self-selection as well as Canadian immigration policies - immigrants to Canada tend to be healthier than the native-born population at the time of their arrival in the country [5,6].

An important dimension to the Canadian literature on the healthy immigrant effect is the notion that although the health of immigrants may be better than that of the native-born population at the time of immigration, that advantage is lost over time. Considering the widespread acceptance of the hypothesis that social conditions are central determinants of health [7-9], this pattern of an advantage that is lost tells us a great deal about the quality of the social fabric and its effect on health, particularly if it describes the experiences of a wide range of immigrant groups across myriad health outcomes.

**Figure 1:**
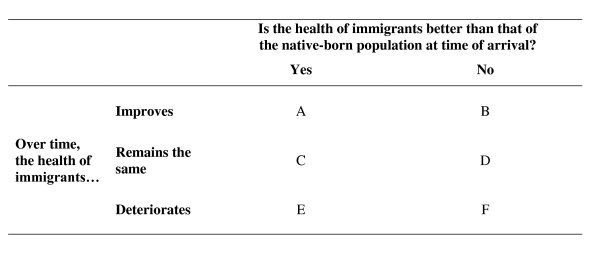
A typology of immigrant health outcomes

A pattern of an initial advantage that is lost over time is one of the six different possible immigrant health transitions described in figure 1. Three of the scenarios (A, C, and E) describe situations where the health of immigrants is better than that of the native-born population - what many studies in this area define as the 'healthy immigrant effect'. Three (B, D, and F) describe situations of an initial disadvantage.

Options B, D, and F fall within the 'sick immigrant' paradigm [4], a perspective that sees immigrants representing a health burden. Whilst empirical research has rejected the validity of this paradigm, it nevertheless still shapes anti-immigration ideologies, often interacting with ideas that immigrants take jobs away from the native-born population. Outcome E (a more nuanced definition of the 'healthy immigrant effect' that is used by many studies in this area; it describes an initial advantage that is lost over time) has been the subject of most recent empirical studies on the health of immigrants to Canada in the past twenty years and is the focus of this paper. The first criterion (an initial advantage in health status in comparison to the native-born population) may be attributable, at least in part, to a self-selection process and the federal government's immigration policies [5,10,11]. The second criterion (the loss of this advantage and deterioration in health status) makes this one of the least desirable outcomes in the typology and would represent a particularly surprising pattern, given Canada's relatively advanced economic standing and the availability of health care services.

If a pattern of deterioration in health status is widespread among immigrants, and if that pattern cannot be explained by compositional characteristics of the immigrants themselves such as aging, 'convergence' factors such as changing patterns of nutrition, or environmental/contextual factors [4], this situation would represent a damning indictment of the quality of the social fabric in Canada. These findings potentially hold very important policy implications, and add further complexity to the study of the social determinants of health.

However, for these implications to be fully understood, we need a better understanding of (a) which health conditions show transition effects and which do not; (b) how immigrants of different ethnicities or 'racialized identities' [12] vary in their health transitions; (c) how health transitions are influenced by socioeconomic status; and (d) how compositional and contextual factors [13] interact to affect the health of immigrants. Analysis of these themes may also indicate differences by age, immigrant type (family-class, economic-class, or refugee), and gender [4,5]. This article provides a systematic review of published empirical studies on the health transitions of immigrants to Canada, highlighting the state of current knowledge on the above points. Based on this review, methodological challenges facing this area of research are critically examined and future directions are identified.

### Review of Empirical Studies

The Medline, Web of Science, SocIndex, and Sociological Abstracts databases were searched in April 2010 for articles that included "immigrant", "health", and "Canada" (or associated terms) in the title, keywords, or abstract. Relevant English-language articles were collected, and their reference sections were examined for other articles related to the present study. Recently published reviews [4,5,11,14] and grey literature reports [15-18] were also examined for relevant empirical studies. Studies suggested by the anonymous reviewers were also considered. Studies needed to meet the following inclusion criteria: (1) must report on empirical analyses which involves a comparison of immigrants and the Canadian-born population or specifically tracks changes in immigrant health over time in Canada, (2) must specify the data source, study population, sample size, and method of analysis, (3) must quantify a health outcome as the dependent variable, and (4) and must have been published since 1990. In total, 1,916 articles were identified as potentially relevant. Articles were excluded if they did not present empirical results of a health outcome, or if the title and abstract made it clear that the paper did not fulfill the inclusion criteria. Collectively, the searches resulted in a total of 51 studies that met these inclusion criteria. These are summarized in table 1.

**Table 1:** Empirical studies of the health transitions of immigrants to Canada

	Dataset/Study	Central Research Question	Health measure	Analysis	Results
**2010**					

Creatore et al [49]	Ontario Diabetes Database	Is there a relationship between length of time in Ontario and age- and sex-related prevalence of diabetes mellitus?	Diagnosis of diabetes mellitus	Logistic regression	For both men and women, time since immigration displayed a gradient-like pattern with diabetes. Income gradient also detected.
De Maio and Kemp [20]	LSIC 2001-2005	To what extent does the health of immigrants deteriorate, and how is this affected by visible minority status and the experience of discrimination?	Self-assessed general health and mental health	Logistic regression	Immigrants' health status deteriorated over the 4-year span of the survey; visible minorities and immigrants that experienced discrimination were most likely to experience worsening health. Some evidence of patterning by socioeconomic status.
Saposnik et al [50]	PRESARIO	Are recent immigrants to Ontario at a higher risk of premature stroke than long-term residents?	Hospitalization for acute stroke before the age of 65.	Cox proportional hazards model	New immigrants are at a lower risk of premature acute stroke than long-term residents. Controlling for income and the availability of health care services does not explain the pattern.
Saposnik et al [51]	MARIO	Are recent immigrants to Ontario at a higher risk of myocardial infarction than long-term residents?	Hospitalization for acute myocardial infarction	Cox proportional hazards model	New immigrants are at a lower risk of acute myocardial infarction than long-term residents. Controlling for income and the availability of health care services does not explain the pattern.
Stafford et al [58]	CCHS 2000/01	How do rates of psychological distress compare between immigrants and the Canadian-born population? And does this vary by visible minority status and the contextual effect of immigrant density?	Depression via CIDI-SF MD	Multilevel logistic regression	Immigrants were less likely to report depression than the Canadian-born population. As the percentage of immigrants in a region increased, the likelihood of depression for immigrants/visible minorities decreased but it increased for whites.
Urquia et al [72]	Ontario Discharge Abstract Database	Is there a relationship between length of time in Canada and adverse birth outcomes?	Preterm and small-for-gestational age births	Multilevel logistic regression	A healthy immigrant effect was observed for risk of preterm birth, but not for small-for-gestational birth outcomes. Long-term immigrants at higher risk of preterm birth than the Canadian-born population.

**2009**					

Chiu et al [64]	Survey of homeless immigrants in Toronto in 2004	Is the healthy immigrant effect generalizable to highly marginalized populations?	Number of chronic conditions, Addiction Severity Index (ASI), and SF-12 health survey	Logistic, Poisson, and linear regression	After adjusting for demographics and lifetime duration of homelessness, recent immigrants hold a health advantage over non-recent immigrants and native-born respondents.
Lear et al [52]	Multi-Cultural Community Health Assessment Trial	Is time since immigration related to the development of sub-clinical atherosclerosis?	Sub-clinical atherosclerosis	Linear regression	Immigration is associated with an increased risk of sub-clinical atherosclerosis. Over time, immigrants lost an initial advantage and their risk surpassed that of the Canadian-born.
Moore et al [71]	Québec Birth Registry Data 2000	Does the association between maternal birthplace, socioeconomic status, and low birth weight vary across immigrant groups and the Canadian-born population?	Low birth weight	Logistic regression	Mixed results, with the strength and direction of the relationship between immigrant status and low birth weight varying by maternal birthplace. Socioeconomic status displayed paradoxical results for some groups, with high SES South Asian- and Caribbean-born mothers having a higher likelihood of low birth weight deliveries than their low SES counterparts.
Newbold [19]	LSIC 2001-2005	Is a deterioration of immigrant's health detectable in the short-term? Does this vary by immigrant category (economic, family reunification, and refugee)?	Self-assessed health	Logistic regression and survival analysis	Signs of worsening health among immigrants may be found very quickly (within 2 years). Refugees are most likely to transition to poor health.
Schaffer et al [62]	CCHS 2002	Do immigrants differ from the native-born population with respect to bipolar disorder (BD)? And among BD subjects, what factors predict mental health service and 12-month psychotropic medication use?	Self-reported BD (definition based on DSM-IV) and Kessler Psychological Distress Scale (K10).	Direct calculation of BD prevalence	Weighted lifetime prevalence of BD is lower among immigrant, compared to native-born, respondents. Among BD subjects, immigrants were less likely to report use of mental health services and had lower (but not significant) rates of psychotropic medication use.
Setia et al [55]	NPHS 1994 - 2006	Does the BMI of immigrants converge with that of the native-born population?	Body mass index	Linear random effects modelling	White male immigrants were the only sub-group to converge to the BMI of the native-born population; other immigrant sub-groups held advantage.
Veenstra [12]	CCHS 2003	Does immigrant status explain the health effects of racialization?	Self-reported diagnosis of diabetes and hypertension, self-assessed health status	Logistic regression	Statistically significant differences between White and 'other' racialized groups exist, even after controlling for the healthy immigrant effect.

**2008**					

Auger et al [70]	Extracted from Quebec Birth Registry 1997-2001	Does a mother's educational attainment interact with immigrant status to influence birth outcomes?	Birth outcomes: small for gestational age, low birth weight, and preterm birth.	Multilevel logistic regression	The influence of immigrant status on birth outcomes differed by educational attainment; immigrant mothers with university education were at higher risk of adverse outcomes. Healthy immigrant effect limited to mothers with low educational attainment.
Kobayashi et al [40]	CCHS 2000/01	Does the healthy immigrant effect differ among Canadians of different ethnocultural origins?	Self-assessed health status, Health Utilities Index, activity restriction	Logistic and linear regression	Visible minority immigrants tend to have better health than their Canadian-born counterparts, with the exception of Chinese and South Asian respondents, for whom the Canadian-born have better health. However, the health of foreign-born and Canadian-born respondents converges after controlling for sociodemographic, socioeconomic, and lifestyle variables.
Stewart et al [67]	Original survey in 10 hospitals (Montreal, Toronto, Vancouver)	Is postpartum depression higher among immigrant women than among Canadian-born women?	Postpartum depression (Edinburgh Postnatal Depression Scale)	Logistic regression	Immigrants, asylum seekers, and refugees were significantly more likely than Canadian-born women to achieve scores reflective of higher risk of postpartum depression.

**2007**					

Leung et al [84]	Original survey of Chinese and white adults in Calgary	Do different measures of health status perform consistently across ethnic populations?	Self-assessed physical and mental health, EQ-5D, number of chronic conditions	Logistic and linear regression	Recent Chinese immigrants were healthier than Canadian-born white population in terms of number of chronic conditions and EQ-5D score. Opposite results when using self-assessed health status and other subjective measures of health.
Mechakra-Tahiri et al [66]	Québec Longitudinal Study of Child Development	Does postnatal depression vary between immigrants (from minority or majority groups) and Canadians?	Self-assessed health, postnatal depression (CES-D)	Logistic regression	Prevalence of high depressive symptoms were highest among immigrants from minority groups, followed by Canadian-born mothers and finally immigrants from majority groups.
Ray et al [69]	RIPPLES	Does the healthy immigrant effect apply to the risk of placental disorders?	Maternal placental syndrome (diagnosis of pre-eclampsia or eclampsia, placental abruption or placental infarction)	Logistic regression	Risk of maternal placental syndrome displays a gradient pattern with months since immigration; healthy immigrant effect is present and diminishes over time.
Smith et al [61]	CCHS 2001/01	How do rates of depression compare between immigrants and the Canadian-born population? And does the relationship interact with income?	Depression via CIDI-SF MD	Logistic regression	Income may interact with gender and immigrant status as a predictor of depression. Male low-income recent immigrants appear to have lower levels of depression than male mid- to high-income recent immigrants.

**2006**					

Newbold [21]	NPHS 1994/95 - 2000/01	Does the healthy immigrant effect apply to the presence, number and type of chronic conditions?	Presence (yes/no), number and type of chronic conditions (any, cardiovascular disease, asthma, arthritis, and diabetes)	Logistic regression, proportional hazard modelling	Support for healthy immigrant effect. Arrival cohort also has a significant effect on chronic conditions. Equalization trend in general with immigrants moving toward native-born health status over time.
Newbold and Filice [33]	CCHS 2000/01	Does the health of older immigrants (aged 55+) display the healthy immigrant effect?	Self-assessed health, Health Utilities Index, presence, number and type of chronic conditions (heart disease, arthritis, asthma, diabetes, cataracts, cancer, emphysema)	Logistic regression	No differences in health status between Canadian-born and foreign-born Canadians aged 55 and older
Sword et al [68]	Original survey from 5 hospitals in Ontario	Do patterns of postpartum health differ between immigrant and Canadian-born women?	Self-assessed health, postpartum depression (4 weeks after delivery)	Chi-square analysis	Immigrant women reported lower overall health and were more likely to indicate possible postpartum depression. Time since immigration was not taken into account.
Zungunegui et al [30]	Health and Social Survey of Quebec 1998, Census of Population and Housing, Police Data	Does community-level unemployment influence the health of immigrants differently than that of the Canadian-born?	Self-assessed health, psychological distress, obesity	Multilevel analysis	At the individual level, no differences between immigrants and non-immigrants in terms of health outcomes. However, in areas of high unemployment immigrants were found to have poorer health in comparison to non-immigrants.

**2005**					

DesMeules et al [73]	Longitudinal Immigration Database, Canadian Mortality Database 1980 - 1998	Do mortality patterns differ between immigrants, including refugees, and the Canadian-born population?	All-cause and disease-specific mortality rates	Poisson regression	In general, immigrants presented lower all-cause mortality than the Canadian-born population. Some cause-specific mortality rates (stroke, diabetes, AIDS and hepatitis) were higher among some immigrants groups, indicating heterogeneity in the healthy immigrant effect.
McDonald & Kennedy [53]	NPHS 1996 and CCHS 2001/01	Is immigration to Canada associated with unhealthy weight gain?	Overweight and obesity	Probit regression	On average, immigrants are less likely to be obese or overweight than the Canadian-born population at the time of their arrival. This advantage is lost over time - but this varies by the ethnicity of the immigrant.
Newbold [28]	NPHS 1994/95 - 2000/01	Do immigrants differ from the native-born population in terms of self-assessed health? Is one group more likely to experience a transition to poor health than the other?	Self-assessed health	Logistic regression, proportional hazard modelling	Mixed support for the health immigrant effect, with immigrants and the native-born equally likely to self-asses their health as poor. Native-born less likely to transition to poor health over time.
Newbold [97]	NPHS 1994/95 - 2000/01	How does the health of immigrants to Canada deteriorate over time?	Self-assessed health, presence of chronic conditions	Logistic regression, proportional hazard modelling	Declining health status of recent arrivals regardless of health outcome used in the analysis. Female immigrants and immigrants with low incomes were at greatest risk of transitioning to poor health.
Ng et al [22]	NPHS 1994/95 - 2002/03	What factors may contribute to changes in immigrants' health after their arrival in Canada?	Self-assessed health, weight gain, along with health care utilization and health-related activities (smoking, physical activity)	Proportional hazard modeling	Immigrant health converges with the host population. Immigrants most likely to have a decrease in self-assessed health status are of non-European origins. Likelihood of deterioration also related to socio-economic status.
Wu and Schimmele [57]	NPHS 1996/97	Does the healthy immigrant effect apply to the risk of depression?	Number of depressive symptoms and experience of major depressive episode	Generalized linear modeling	Depression rates are lower for immigrants than the native-born upon arrival but increases soon after arrival.
Wu and Schimmele [31]	NPHS 1996/97	Does immigrant status help to explain racial/ethnic differences in health?	Self-assessed health status, functional health	Linear and cumulative logit regression	No clear pattern between racial/ethnic identity and health inequities. Immigrant status not a significant predictor.

**2004**					

Gee et al [39]	CCHS 2000/01	Does the healthy immigrant effect apply in mid-age and older-age?	Self-assessed health status, Health Utilities Index, self-reported activity restrictions	Logistic regression	Healthy immigrant effect is present among mid-life (45 - 64 years) immigrants. Immigrants aged 65 and older appear to have worse health than Canadians aged 65 and over, but this disadvantage disappears after controlling for other independent variables.
Malenfant [76]	Canadian Vital Statistics Data Base	Do rates of suicide differ between immigrants and the Canadian-born population?	Age-standardized suicide rate	Comparison of crude and standardized rates	Suicide rates for the immigrant population is about half that of the Canadian population. Increases in age are associated with increases in suicide among immigrants, whereas the opposite holds for the Canadian-born.
McDonald & Kennedy [29]	NPHS 1996 and CCHS 2000/01	Does the healthy immigrant effect apply to self-assessed health and the presence of chronic conditions?	Self-assessed health status, presence of chronic conditions	Probit regression	Support for healthy immigrant effect for recent immigrant arrivals. Advantage continues, but to a lesser extent with time since migration. Significant evidence of a cohort effect for immigrant health, though it is insufficient to explain convergence of immigrant health status to the native-born health status.
Vissandjee et al [43]	CCHS 2000/01	Does the healthy immigrant effect remain significant after controlling for ethnicity?	Self-assessed health status, presence of chronic conditions	Logistic regression	Female immigrants with less than 2 years in Canada are least likely to self-report poor health, but this advantage may be lost to the point where female immigrants who have been in the country for more than 10 years are the most likely to self-report poor health.

**2003**					

Newbold & Danforth [41]	NPHS 1998/99	What differences exist in the health of immigrants and the native-born population? Do these differences reflect the effects of social determinants of health?	Self-assessed health status, presence of chronic conditions, Health Utilities Index	Logistic and linear regression	Overall, immigrants were found to have poorer health than non-immigrants. However, the healthy immigrant effect was observed when the period of arrival was controlled for. Those who had been in the country for more than ten years reported worse health than recent immigrants.
Kobayashi [44]	NPHS 1996/97	Does immigration status intersect with ethnicity to influence health status in mid- to later-life Canadians?	Presence of chronic conditions	Logistic regression	Significant differences in health status based on time since immigration and country of birth, with recent immigrants from Asia and non-European countries displaying a lower odds of having at least one chronic condition than Canadian-born respondents.

**2002**					

Ali [56]	CCHS 2000/01	Does the healthy immigrant effect apply to depressive symptoms and alcohol dependence?	Depression and alcohol dependence, both CIDI assessed	Logistic regression	Immigrants were found to have lower rates of depression and alcohol dependence than the Canadian-born population. This advantage diminishes as length of residence in Canada increases.
Beiser et al [63]	NLSCY 1994/95	Does the healthy immigrant effect apply to the mental health of immigrant children?	Psychometric scales of emotional and behavioural problems	Linear regression	Foreign-born children had lower levels of emotional and behavioural problems than Canadian-born children, despite being twice as likely to live in poor families.
Pérez [45]	CCHS 2000/01	Does the healthy immigrant effect apply to chronic conditions?	Presence of chronic conditions	Logistic regression	Both male and female immigrants had lower odds of reporting chronic conditions than the Canadian-born respondents, and these odds increased with time since immigration. Male immigrants had lower rates of heart disease than native born males. Women immigrants had lower rates of cancer than the Canadian-born respondents. With respect to diabetes and high blood pressure, no differences were observed.
Payne et al [74]	Canadian Mortality Database 1980-1998	What factors may be associated with mortality for immigrant women?	Standardized mortality ratios	Poisson regression	Significantly increased risk of mortality among certain groups of women, including among refugees versus non-refugees.

**2001**					

Kopec et al [36]	NPHS 1994/95	What differences exist in health status between cultural groups defined by place of birth and language?	Health Utilities Index	Logistic regression	Mixed support for the health immigrant effect, with some (but not all) immigrant groups reporting higher levels of health than Canadian-born respondents.

**2000**					

Dunn & Dyck [46]	NPHS 1994/95	Do social determinants of health influence the health of immigrants and the Canadian-born population in different ways?	Self-assessed health status, presence of a chronic condition	Logistic regression	Ambiguous results with no consistent patterns between socio-economic characteristics and health status or immigration characteristics and health status. Socio-economic factors may be more important for immigrants than non-immigrants.
Laroche [10]	GSS 1985, 1991	Does the health status of immigrants differ from that of the Canadian-born population?	Self-assessed health status, long-term activity limitation	Probit regression	Health status of immigrants does not differ significantly from that of the Canadian-born population. However, immigrants are less likely to report long-term activity limitation than the native-born.
Wang et al [47]	NPHS 1994	Do arthritis rates differ between immigrants from Asia, Europe/Australia, and the Canadian-born population?	Arthritis or rheumatism	Logistic regression	Age-sex adjusted rates of arthritis were lowest for immigrants from Asia, followed by immigrants from Europe/Australia and finally, Canadian-born respondents.

**1999 and earlier**					

Cairney & Ostbye [54]	NPHS 1994	Is time since immigration associated with excess body weight?	Self-reported height and weight	Logistic and linear regression	Prevalence of excess weight increases after immigration for both men and women. Adjusted models indicate the healthy immigrant effect may apply to women and Asian men.
Chen et al [48]	NPHS 1994/95	Do immigrants differ from the Canadian-born population with respect to asthma?	Asthma	Logistic regression	Immigrant status and household income were significant predictors of asthma prevalence for both men and women.
Chen, Wilkins, & Ng [75]	Canadian Vital Statistics Data Base 1985-87, 1990-92, Census 1986, 1991, Health and Activity Limitation Survey 1986-87, 1991	Does life expectancy vary between immigrants and the Canadian-born population?	Crude, disability- and dependency-adjusted life expectancy	Prevalence estimates	Support for the healthy immigrant effect. Immigrants, particularly those from a non-European country, had a longer life expectancy and more years free of disability and dependency
Chen, Ng, & Wilkins [42]	NPHS 1994/95	Does the healthy immigrant effect apply in Canada?	Presence of a chronic condition, disability, and health-related dependency	Prevalence estimates	Non-European immigrants in particular and immigrants in general were healthier than non-immigrants for chronic conditions. Prevalence of chronic conditions increases with time since immigration.
Noh and Avison [65]	Korean Mental Health Study 1990-91	How are stressors and psychological/social resources related to psychological distress among Korean immigrants?	Psychological distress based on CES-D (translated)	Linear regression	Number of years in Canada does not appear to influence levels of psychological distress.
Parakuluam et al [98]	GSS 1985, Census 1981	Does the health status of the Canadian-born differ from that of foreign-born residents?	Derived 'healthfulness' index (presence of chronic conditions, utilization of health services, activity limitation or disability)	Descriptive statistics	Overall, immigrants are healthier than native-born Canadians.

*Abbreviations: *BD - Bipolar disorder; BMI - Body mass index; CCHS - Canadian Community Health Survey; CIDI-SF MD - Composite Diagnostic Interview Schedule Short Form for Major Depression; CES-D - Center for Epidemiologic Studies Depression Scale; GSS - General Social Survey; LSIC - Longitudinal Survey of Immigrants to Canada; MARIO - Myocardial Infarction Associated with Recency of Immigration to Ontario; NLSCY - National Longitudinal Survey of Children and Youth; NPHS - National Population Health Survey; PRESARIO - Premature Stroke Associated with Recency of Immigration to Ontario; RIPPLES Recent Immigrant Pregnancy and Perinatal Long-Term Evaluation Study

The majority of studies in the review analysed data from either the National Population Health Survey (NPHS) or the Canadian Community Health Survey (CCHS) - two of Statistics Canada's major social surveys. These datasets can be used to compare health outcomes between immigrants and the Canadian-born, with the additional advantage of being able to differentiate recent immigrants (less than 10 years in the country) from long-term immigrants (10 or more years in the country). With some exceptions, the CCHS-based literature indicates substantial differences between the health of immigrants and the Canadian-born population. However, due to their reliance on cross-sectional data, these studies do not provide an analysis of the actual health transitions of individual immigrants. Indeed, whilst CCHS-based studies enable comparison of new-versus long-term immigrants, the interpretation of observed differences may confound cohort and time effects [4]. Immigration patterns have changed substantially in the recent history of the country, and it is difficult to infer a pattern by comparing the health status of recent immigrants with the health of immigrants who have been in the country for 20 or more years. Longitudinal data sources, in this case either the Longitudinal Survey of Immigrants to Canada [LSIC; see [19,20]] or the longitudinal component of the NPHS [21,22] offer particular advantages in this respect.

### Patterns of Health Transitions

The health of the immigrant population in Canada has been investigated using a wide range of measures, including self-assessed health status, diagnosed chronic conditions, obesity, mental health, birth outcomes, and mortality. The studies from table 1 are summarized by outcome measure in table 2 and subsequently discussed in detail below.

**Table 2:** Overview of results by outcome measure

	Number of studies	Results/Emerging Themes
Self-assessed health status		
- Likert scale	18	Using social surveys such as the NPHS, the CCHS, and the LSIC, these studies have shown (with a few exceptions) that the health of immigrants is better than that of the Canadian-born population at time of arrival and that this advantage is lost over time.
- Health Utilities Index (HUI)	5	Studies using the HUI detected the healthy immigrant effect among mid-life (aged 45-64 years of age) immigrants and among visible minorities. Results from HUI studies also emphasize the heterogeneity found among immigrants, with significant differences in the reporting of pain, emotional function, and cognitive function among different immigrant groups.
Chronic conditions		
- Presence of chronic conditions	14	Overall support for the healthy immigrant effect, with some exceptions - likely attributable to conflation of different kinds of conditions into a dichotomous outcome (1 = yes, 0 = no).
- Arthritis	1	Age-sex adjusted rates of arthritis are lower among immigrants than the Canadian-born population. Effect of duration of residence is unknown.
- Asthma	1	Prevalence of asthma is lower among immigrants than the Canadian-born population. Effect of duration of residence is unknown.
- Cardiovascular diseases	3	Immigrants enjoy lower risks of premature acute stroke and myocardial infarction, even after adjusting for income and availability of health care services. Duration of residence is associated with sub-clinical atherosclerosis.
- Diabetes	5	Mixed results. Some indication that immigrants enjoy lower rates of diabetes, even after adjusting for income, educational attainment, age, and other factors. However, new data shows that recent immigrants, particularly women and immigrants of South Asian and African origin, may be at a higher risk for diabetes mellitus compared to long-term residents.
Obesity	5	On average, immigrants are less likely to be obese or overweight than the Canadian-born population at the time of their arrival. This advantage is lost over time - but this varies by the ethnicity of the immigrant. Most recent findings suggest that white male immigrants are most likely to converge to Canadian-born rates of obesity; other immigrant groups tend to enjoy lower BMI even over time.
Mental health		
- Depression/distress/other	10	Depression and other mental health issues may be less prevalent among immigrants than the Canadian-born population. However, this advantage diminishes as length of residence in Canada increases. Living in areas with a high density of immigrants may help immigrants to retain this advantage. Immigrants who experience discrimination may be more likely to report worsening health. Income may interact with gender as a determinant of mental health, with mid-/high-income immigrants faring worse than expected given their income status.
- Postpartum depression	3	Postpartum depression may be more prevalent among immigrant women than Canadian-born women. However, studies in this area have not controlled for time in Canada; differences between recent- and long-term immigrants may be confounded in the results.
Birth outcomes	4	Time in Canada may be associated with an increased risk of placental disorder and preterm birth but not for small for gestational age births. Highly educated immigrant women experience worse than expected outcomes.
Mortality	3	Some indication of lower mortality risk among immigrants overall, but with considerable heterogeneity in patterns by cause and between type of immigrant (economic, family, refugee)
Suicide	1	Suicide rates for the immigrant population is about half that of the Canadian population. Increases in age are associated with increases in suicide among immigrants, whereas the opposite holds for the Canadian-born.

#### Self-assessed health status

Self-assessed health status was the most commonly used outcome measure in these studies, with 18 of the 51 articles relying on the standard 'In general, how would you rate your health?' question answered on a five-point Likert-type scale. This question is commonly used in medical sociology and social epidemiology, and has been found to be highly predictive of actual health status, including subsequent morbidity [23] and mortality [24,25]. Although some researchers have raised questions regarding the validity [26] and reliability [27] of self-reported health status measures, they remain an important and useful part of the methodological toolbox for inequality researchers.

Veenstra's [12] analysis of the CCHS is indicative of this type of study. Controlling for racial/cultural identification, educational attainment, household income, urban/rural residence, and region of residence, he found that recent immigrants (in Canada less than 5 years) had a significantly lower odds of reporting poor health compared to the Canadian-born (OR = 0.51, p < 0.001). Consistent with the deterioration trajectory described in cell E of figure 1, immigrants who have been in the country for more than 10 years reported a higher odds of poor health, compared to the Canadian-born (OR = 1.15, p < 0.001). Veenstra's results echo those of Ng et al [22], who found that immigrant self-assessed health converges with that of native-born Canadians over time using the longitudinal component of the NPHS. Ng et al's findings also begin to tease apart the heterogeneity of immigrant's experiences; immigrants of non-European origins were most likely to have a significant decrease in self-assessed health status. Deterioration of health status was also closely associated with low education and low household income. Cohort period may also be important; Newbold's [28] analysis of the NPHS indicates that immigrants who came to Canada between 1990 and 1994 were less likely than the native-born to rank their health as poor in 1994/95, but by the 2000/01 longitudinal follow-up, this advantage had been lost altogether. Differences consistent with the healthy immigrant effect were also found by McDonald and Kennedy [29], in their pooled cross-sectional analysis of the CCHS and the NPHS.

In recently published studies of self-assessed health data from the LSIC, Newbold [19] and De Maio and Kemp [20] found support for the notion of a trajectory of deterioration in health. Newbold's analysis indicates that refugees are the most likely to transition to poor self-assessed health, whilst De Maio and Kemp indicate that visible minorities and immigrants who experienced discrimination or unfair treatment after settling in Canada are most likely to experience a decline in self-assessed health.

Most of the studies using self-assessed health status as the outcome variable support the notion that the health of immigrants is better than that of the Canadian-born population at time of arrival but that this advantage is lost over time (see table 1). There are, however, a few exceptions [10,21,30,31] - situations where the 'healthy immigrant' effect is not present. This is to be expected; there is tremendous heterogeneity among immigrants to Canada, and their experiences in the country do differ [32]. For example, no difference in self-assessed health status was found in the 55 and over age group [33], suggesting that the factors driving the health transitions of immigrants may be life-stage dependent. At the same time, not all of the studies incorporated the length of time an immigrant has been in the country, and instead modeled the effects of immigrant status as a yes/no response. This likely had the effect of obscuring differences within immigrant respondents.

Whilst studies of self-assessed health status form an integral component of this research literature, they suffer from an important methodological weakness: we know little of how immigrants' expectations of their health changes after settling in Canada. It could be that part of the adaptation process involves developing new thresholds for what constitutes health and illness [19-21]; this would imply that instead of deterioration in health, changes in self-assessed health status may be reflective of increased expectations that may be associated with life in Canada. Indeed, the sociological literature emphasizes the relative nature of self-reported health status, with individuals judging their health in comparison to complex reference groups [34,35]. However, little is known about this phenomenon among immigrant groups.

Five of the 51 studies utilized the Health Utilities Index (HUI), a multidimensional measure of functional health [36]. It is a weighted index of an individual's health based on eight self-reported core attributes: vision, hearing, speech, emotion, dexterity, cognition, and pain [37,38]. Studies using the HUI detected the healthy immigrant effect among mid-life (45-64 years of age) immigrants [39] and among visible minorities [40]. Results from HUI studies also emphasize the heterogeneity found among immigrants. For example, Kopec et al [36] found significant differences in the reporting of pain, emotional function, and cognitive function among different immigrant groups. Newbold and Filice [33] report that new immigrants conform to the healthy-immigrant prediction and report better HUI scores than the Canadian-born population; consistent with the notion of deterioration (but not explicitly tested as their CCHS data is strictly cross-sectional), immigrants with more than 10 years in the country experienced worse HUI scores [see also [41]].

#### Presence/Number of Chronic of Conditions

Strong support for the healthy immigrant effect is found in studies that focused on chronic diseases. The majority of these studies operationalised the presence of chronic diseases as a dichotomous outcome, distinguishing cases were a diagnosis for any chronic disease had been made by a healthcare professional and cases where that had not been the case. For example, respondents in the NPHS were asked "Do you have heart disease diagnosed by a health professional?". The survey also asked about other chronic disease diagnoses, including arthritis, diabetes, and high blood pressure. The CCHS has followed a similar strategy. In a few studies key conditions were examined separately, but the overall trend has been to aggregate any chronic condition into a positive outcome on a dichotomy. McDonald and Kennedy's [29] study offers a slight variant on this approach, because they distinguished "Type A" conditions (generally not life threatening: asthma, back pain, high blood pressure, allergies, etc) from Type B conditions (including heart disease, cancer, and diabetes) and created separate dichotomous outcome variables for each condition.

Newbold's [21] longitudinal analysis of the NPHS supports the healthy immigrant effect for chronic conditions. His results indicate that immigrants are less likely to report having a chronic health condition than the Canadian-born population, but that this advantage is lost over time through a pattern of convergence. Other studies of the NPHS [42] as well as the CCHS [43-45] support that assessment. However, Newbold and Filice [33], in an analysis of older immigrants (aged 55 and over) found no differences in chronic conditions between immigrants and their Canadian-born counterparts, and Dunn and Dyck found "no obvious, consistent pattern of association between socio-economic characteristics and immigration characteristics on the one hand, and health status on the other" [46] in the NPHS. Similarly, Laroche's [10] analysis of the GSS found no significant differences between immigrants and the Canadian-born when using a dichotomous outcome contrasting respondents with a diagnosis of heart trouble, diabetes, respiratory problems, or arthritis from those without. It may be that these studies, by virtue of conflating a number of diseases with distinct etiologies and epidemiologies, diminished the statistical power of their analyses. An alternative approach, one that distinguishes between different kinds of chronic diseases, is perhaps preferable.

In contrast to the generalized approach of the studies above, a few studies developed separate models for specific chronic disease outcomes, including diabetes, cancer, and hypertension [12,45], arthritis [47], and asthma [48]. Using these more specific measures of chronic disease, Wang et al [47] found that age-sex adjusted rates of arthritis were lower among immigrants than the Canadian-born population and Chen et al [48] found similar results for asthma. Neither study controlled for duration of residence and therefore do not indicate if the health advantage of immigrants diminished over time. Veenstra's [12] analysis of the CCHS suggests that over time in Canada, an immigrant's odds of developing diabetes and hypertension increase, even after controlling for racial/cultural identification, educational attainment, household income, and region of residence. Creatore et al [49], in an analysis of the Ontario Diabetes Database, support Veenstra's analysis and show that recent immigrants, particularly women and immigrants of South Asian and African origin, may be at a higher risk for diabetes mellitus compared to long-term residents of the province of Ontario. Odds of being diagnosed with diabetes were found by Creatore et al to be related in gradient-like ways with time since immigration and low income.

Three recent studies have used clinical measures/hospitalization records to examine the healthy immigrant effect and cardiovascular diseases. The results of these studies indicate that immigrants enjoy lower risks of premature acute stroke [50] and myocardial infarction [51], even after adjusting for income and availability of health care services. Lear et al's [52] analysis of 460 immigrants and 158 Canadian-born subjects without pre-diagnosed cardiovascular disease (CVD) shows that immigration is associated with an increased risk for atherosclerosis (assessed in the study by carotid artery ultrasound). Their analysis shows that whilst immigrants as a whole have a lower burden of sub-clinical atherosclerosis than does that Canadian-born population, this advantage is lost over time. One indicator (and a risk factor for CVD), intima-media thickness (IMT), increased by 2% for every 10 years in Canada. Their analysis showed not just a convergence but overshoot in IMT values. Lear et al note: "...as time since immigration increased, the IMT in immigrants surpassed that of the non-immigrants independent of age and other confounders. This would suggest that while immigrants may be as healthy, or healthier than non-immigrants in Canada, they experience a rapid increase in sub-clinical atherosclerosis compared to non-immigrants" [52].

The adoption of so-called "Western" eating habits and lifestyles has been suggested as a driver of these kind of transitions. Yet Lear et al's analysis also included immigrants from Europe, and they too experienced an increase in IMT values as duration of residence in Canada lengthened. For Lear et al, this suggests that immigration itself, and not the adoption of Western risk factors, is the culprit. However, other than noting that this is likely not a "Westernization" narrative, Lear et al do not elucidate the drivers of the health transitions they document, noting that the underlying reasons for their findings remain unknown.

#### Obesity

An important subset of studies in this area has compared the body mass index of immigrants to the Canadian-born population. The findings suggest that on average, immigrants are less likely to be obese or overweight than the Canadian-born population at the time of their arrival [22,53]. This advantage is lost over time [54] - but this varies by the ethnicity of the immigrant [53]. One exception to that conclusion is offered by Zunzunegui et al [30]; their multilevel analysis of data from a health survey in Montreal revealed no differences between immigrants and the Canadian-born respondents in terms of their body mass index. However, their analysis did not take into account how long an immigrant has been in the country (except for distinguishing first- from second-generation immigrants). The result may be that Zunzunegui et al's analysis confounds differences between recent and long-term immigrants.

The most recent findings in this area suggest that white male immigrants may be most likely to converge to Canadian-born rates of obesity; other immigrant groups tend to enjoy lower BMI even over time. Setia et al's [55] 12-year longitudinal analysis of the NPHS compared changes in BMI among white immigrants, non-white immigrants, and the Canadian-born. They found that ever after adjusting for duration of residence, non-white immigrants had lower BMI than white immigrants, and that the latter where the only group to converge with the BMI levels of the Canadian-born population. As is the case with a growing set of studies that find heterogeneity in the health transitions of immigrants, Setia et al call into question the generalizability of the healthy immigrant effect, noting that "convergence of BMI to Canadian levels may not be experienced equally by all immigrants to Canada" [55].

#### Mental Health

An important branch of this literature has examined mental health outcomes. Several of these analysed data from the Composite International Diagnostic Interview - Short Form for Major Depression (CIDI-SF MD) instrument that is included in the CCHS. Analyses of these data suggest that immigrants may have lower rates of depression than the Canadian-born population at time of their arrival in the country, but that this advantage diminishes with duration of residence [56,57]. The most recent findings are those presented by Stafford et al [58]; they found that recent immigrants (less than 10 years in Canada) have the lowest prevalence of depression (4.2%), followed by long-term immigrants (6.5%) and the Canadian-born population (8.0%; all crude prevalence data). Unlike the majority of studies in this area, Stafford et al explicitly model contextual effects [13,59]; their multilevel analyses indicate an intriguing pattern: as the percentage of immigrants in a region increased, the likelihood of depression for immigrants/visible minorities decreased but it increased for whites. Drawing on recent work on the protective effect of increasing minority density [60], Stafford et al poignantly question if "visible minorities experience less discrimination and stigma in health regions of higher immigrant density" [58]. However, given the limitations of the CCHS (which does not include measures of discrimination), Stafford et al could not empirically investigate that question.

The notion of discrimination as a driver of immigrant health is perhaps most strongly reflected in De Maio and Kemp's [20] analysis of the LSIC. Using data from all three waves of the longitudinal study, De Maio and Kemp's findings suggests that immigrants who experienced discrimination were most likely to experience worsening self-reported mental health, operationalised as increasing levels of feelings of sadness, depression, and loneliness. Almost 30% of respondents in the LSIC reported experiencing discrimination or unfair treatment because of their ethnicity, culture, race or skin colour, language or accent, or religion. This was associated with an increased odds of experiencing a worsening of self-reported mental health (OR = 2.33, 95% CI = 2.05 - 2.64, controlling for socioeconomic status and demographics).

De Maio and Kemp also report that the likelihood of a deterioration in self-reported mental health status was patterned by socioeconomic status. It was highest among immigrants in the lowest income quartile (OR 2.03, 95% CI = 1.72 - 2.39), followed by respondents in the medium low (OR 1.53, 95% = 1.30 - 1.81) and medium high (OR = 1.30, 95% CI = 1.10 - 1.54; all unadjusted) quartiles, in contrast to immigrants in the highest income quartile. Women were also more likely than men to have experienced a deterioration (OR = 1.53, 95% 1.37 - 1.71; unadjusted). Both effects remained stable as more variables were added to the model.

Using the same wave of the CCHS and the CIDI-SF MD based measured of depression as Stafford et al, Smith et al [61] examined the possible interaction of recent immigration, income, and gender as predictors of depression. Smith et al's findings support the general 'healthy immigrant' pattern, with the prevalence of depression being lowest among recent immigrants (5.2%), followed by long-term immigrants (9.5%), and finally Canadian-born respondents (10.1%). Their multivariate analyses detected an important interaction effect, with male low-income recent immigrants appearing to have lower levels of depression than male mid-/high-income recent immigrants. In contrast, female low-income recent immigrants fare poorly compared to middle- and high-income recent immigrants. In other words, a differential income effect for male and female recent immigrants may exist.

Other studies on mental health have focused on specialized issues, such as bipolar disorder [62], the mental health of immigrant children [63], the mental health of homeless immigrants [64], and psychological distress [30,65]. Schaffer et al analysed data from the CCHS 1.2, which focused on mental health and well-being, and found that lifetime prevalence of bipolar disorder was significantly lower among immigrant groups compared to the Canadian-born population (1.5% versus 2.3%, p < 0.01). Zunzungegui et al [30] provide further support Stafford et al's argument that context matters as a determinant of immigrant health; in this case, Zunzungegui et al found that in areas of high unemployment, immigrants report higher levels of psychological distress than do non-immigrants. Noh and Avison [65], in a study of Korean immigrants to Toronto, found that psychological distress was not influenced by the number of years an immigrant has been in Canada - a result that is not supported by more recent studies using larger datasets [20,56-58,61].

Three studies have examined postpartum depression. The most informative of these is Mechakra-Tahiri et al's [66] analysis of the Quebec Longitudinal Study of Child Development. They used the 12-item Center for Epidemiologic Studies Depression Scale (CES-D) to compare postnatal depressive symptoms in mothers 5 months after giving birth, comparing according to immigration status. They found that the prevalence of depressive symptoms was highest among immigrants from minority groups (at 24.7%), followed by Canadian-born mothers (11.2%), and finally immigrants from majority groups (8.3%, with majority group describing immigrants from Europe or countries with English ancestry: USA, Australia, or New Zealand). Similar results were presented by Stewart et al [67], who administered the Edinburgh Postnatal Depression Scale (EPDS) to 719 women in 10 hospitals in the main immigrant receiving cities of Toronto, Montreal, and Vancouver. They found that immigrant women had a 3 to almost 5-times increased risk of EPDS scores reflective of possible postpartum depression. Visible minority status was associated with an increased but not statistically significant risk of a high EPDS score (OR = 1.75, 95% CI = 0.95 - 3.21). In general, immigrants, asylum seekers, and refugees were significantly more likely than Canadian-born women to achieve scores reflective of higher risk of postpartum depression. Stewart et al suggest that these findings perhaps indicate a lack of social support, difficulties with language, and/or "unfamiliarity with Canadian life and health care" [67]. These studies echo the results of Sword et al [68], who analysed data from a sample of 1,250 women following the delivery of a healthy infant. They found that immigrant women reported lower overall health and were more likely to indicate possible postpartum depression (indicated via EPDS scores). A limitation of this set of studies is that none controlled for time since immigration and may thereby have confounded differences between recent- and long-term immigrants.

#### Birth Outcomes

A recent development in this area has been the use of birth outcomes as an indicator of the health condition of immigrant women. This is an important extension, as it enables analyses of outcome measures that do not suffer from the validity and reliability concerns that hamper self-assessed questions of health status. It also integrates the research programme on the healthy immigrant effect with questions of maternal health - an important element of any comprehensive discussion of health equity.

The first study of this type was published by Ray et al [69], who questioned if the 'healthy immigrant' effect extended to the risk of placental dysfunction (defined as a diagnosis of pre-eclampsia or eclampsia, placental abruption or placental infarction). They found a gradient-like pattern after adjusting for maternal age, income status, pre-existing hypertension and other related factors. More specifically, their analysis indicated that compared to women who had resided in Ontario for more than 5 years, women who had been in the province less than 3 months had the lowest odds of placental dysfunction (OR = 0.53, 95% CI = 0.47 - 0.61), followed by women who had been in the province 3 - 5 months (OR 0.68 = 0.68, 95% CI = 0.61 - 0.76), 6 - 11 months (OR = 0.67, 95% CI = 0.63 - 0.71), 12 - 23 months (OR = 0.69, 95% CI = 0.66 - 0.73), 24 - 35 months (OR = 0.75, 95% CI = 0.70 - 0.79), 36 - 47 months (OR = 0.75, 95% CI = 0.70 - 0.80), and 48 - 59 months (OR = 0.82, 95% CI = 0.77 - 0.87). Ray et al did not control for race/ethnicity and therefore do not shed light on the health outcomes of visible minorities. Despite that limitation, their study is important in that it "rebuts the common perception that immigrants might consume more health care resources than do long-term residents" [69] and because it was the first study to extend the healthy immigrant research programme into the area of birth outcomes. Their study was followed by investigations of immigrant status and preterm birth (PTB), small-for-gestational age (SGA) birth, and low birth weight (LBW) that revealed underlying paradoxical effects of socioeconomic status [70,71] and emphasized the importance of taking into account the length of time an immigrant has been in the country [72].

Auger et al's analysis of Quebec Birth Registry data demonstrated that the influence of foreign-born status on birth outcomes differs by the educational attainment of the mother. More specifically, low educational attainment was associated in their study with LBW for Canadian mothers (OR 3.20, 95% CI = 2.61 - 3.91) but not foreign-born mothers (OR = 1.14, 95% CI = 0.99 - 2.10). Auger et al also report that foreign-born status was associated with SGA birth, LBW, and PTB in university-educated mothers. They conclude, therefore, that the 'healthy immigrant' effect may extend to mothers with low education but not to mothers with high educational attainment; this group of immigrants may actually experience a greater than expected health burden. Auger et al note that "the mechanisms that underpin the associations reported here are unclear... After entry into Canada, immigrant women of higher education could conceivably experience greater stress adapting to a new living environment" [70]. Their analysis opens up new lines of questioning regarding stress mechanisms associated with social class, and how they interact with immigrant status. Worse than expected outcomes for highly educated immigrant women may indeed be the result of stress mechanisms, but it is unclear why these stress mechanisms would operate in ways that contradict the general gradient produced by social determinants of health. One explanation that does not contradict the predictions based on the social determinants of health is that the findings presented by Auger et al may actually be driven by discrimination and loss of social status, an explanation with some support from Moore et al's [71] analysis of the same data source.

Quebec Birth Registry data were analysed Moore et al [71] and the results add further complexity to the determinants of birth outcomes for immigrant versus native-born mothers. Moore et al found that the strength and direction of the relationship between foreign-born status and LBW varies by maternal birthplace, and perhaps most importantly, that the effect of socioeconomic status also varies by maternal birthplace. In particular, their analysis indicates that high socioeconomic status "does not bring [to immigrants] the same perinatal health-related benefits as it does for Canadian-born mothers" [71]. They note that various mechanisms may account for this effect, including those embedded within socioeconomic discrimination.

The latest study in this area examines immigrants' duration of residence in Ontario and adverse health outcomes. More specifically, Urquia et al [72] compared PTB and SGA births among non-immigrants and immigrants, and examined the influence of length of time in Canada. They report that recent immigrants (women in Canada less than 5 years) had a lower risk of PTB (4.7%) than non-immigrant women (6.2%). Long-term immigrants (women in Canada more than 15 years) had the highest risk of PTB (7.4%). These results, therefore, suggest that not only did 'healthy immigrant' effect extend to PTB, but the patterning of the effects was not a convergence but an overshoot - something that would have been missed if an immigrant's length of time in Canada was not taken into account. Urquia et al also report that, among immigrants, a 5-year increase in time in Canada was associated with an increase in PTB (adjusted OR 1.14, 95% CI = 1.10 - 1.19). Importantly, duration of residence was not associated with an increase in SGA among immigrant women (adjusted OR = 0.99, 95% = 0.96 - 1.02). This suggests that the influence of duration of residence may be outcome-specific. In interpreting their results, Urquia et al offer a plausible explanation for the association of time in Canada and PTB but not SGA, noting that other studies have found that maternal stress is associated with PTB but not with intrauterine growth restriction. This once again brings the focus to psychosocial factors, of which discrimination may be a central dimension.

#### Mortality

A small subset of studies has examined mortality through data from the Canadian Mortality Database [73-75]. These studies give us some indication of a lower mortality risk among immigrants overall, but also indicate the presence of considerable heterogeneity in patterns by cause of death and between types of immigrant (e.g., economic, family, refugee). For example, DesMeules et al found a lower all-cause mortality rate for immigrants than the general population (standardized mortality rates between 0.34 and 0.58), but also detected elevated cause-specific rates among immigrants (in the cases of mortality from stroke, diabetes, liver cancer, and AIDS). These may be conditions where immigrant populations experience particular barriers in accessing effective treatments. The one study published to date on suicide suggests that suicide rates for the immigrant population is about half that of the Canadian population [76].

### Patterns by Ethnicity

Studies in this area have tended to operationalise ethnicity as birthplace (country or region) or through Statistics Canada's 'visible minority' terminology [77]. However, these studies are just beginning to 'unpack' the role of ethnicity as it relates to the health of immigrants [43], and debates continue on conceptual and methodological grounds [78]. Veenstra's [12] analysis of the CCHS is perhaps the most informative work on this issue; building from critiques of 'race' [79,80], his analysis calls for the use of a more theoretically-nuanced term 'racialized identity'. This term recognizes the social construction that underlies any categorization system for race/ethnicity, and allows us to see such systems as historically and contextually specific identities shaped by relations of power and inequality. His analysis indicated that no racial/cultural group achieved better health outcomes than those achieved by White respondents in the CCHS. In other words, health inequities existed in his analysis between respondents of different racialized identities, even after controlling for immigrant status, socio-economic status, and demographics. Veenstra concludes by noting that "it seems reasonable to hypothesize that some of the unexplained health disparities by racial/cultural identification in this dataset reflect the wear and tear of experiences of racism and discrimination in regular encounters with societal institutions and in everyday life" [12].

Along these lines, De Maio and Kemp [20] used the LSIC to examine the health effects of discrimination. Immigrants who reported experiencing discrimination or unfair treatment because of their ethnicity, culture, race or skin colour, language or accent, or religion had higher odds of experiencing a worsening of self-reported mental health (OR = 2.33, 95% CI = 2.05 - 2.64, controlling for socioeconomic status and demographics) and self-assessed general health (OR = 1.17, 95% CI = 1.04 - 1.31, again controlling for socioeconomic status and demographics).

The patterning of health transitions by ethnicity may be dependent on the choice of outcome measures. The studies discussed above [12,20] both used self-assessed health status as the outcome measure and found that 'non-White' respondents fared worse than 'White' respondents. In contrast, Stafford et al [58] found that immigrant and visible minority residents were less likely than the general population to experience depression, as measured in the CCHS through the Composite Diagnostic Interview Schedule, and Setia et al's [55] analysis of the NPHS found that non-white immigrants were most likely to retain an initial advantage in terms of body mass index over the general population as their length of residency in the country increased. Altogether, these findings suggest that ethnicity needs to be conceptualized as an integral component of any analysis of the health of immigrants to Canada - heterogeneity, rather than homogeneity, is likely the pattern at play. However, for this dimension to be fully understood, more research is needed on the interaction between measures of ethnicity and discrimination, and these measures - along with an immigrant's length of residency in Canada - should be incorporated into future analyses.

At the same time, this area of research could benefit from a closer integration with the rapidly growing literature on intersectionality [80]. This concept emphasizes that race/ethnicity and class intersect with gender to produce health whilst at the same time arguing that race/ethnicity, class, and gender should not simply be seen as characteristics of individuals, but should be understood as social relations. Mullins and Schulz [81] argue that traditional epidemiological methods (based on a positivist epistemology and quantitative methodology) will have difficulty incorporating these conceptualizations; that by their very nature and assumptions, these models call for discrete variables that exert independent effects on a dependent variable and are therefore ill-suited to disentangling the complex interactions between race/ethnicity, class, and gender [7]. One of the challenges facing this area of research, therefore, is to develop epistemologically and methodologically nuanced analyses that acknowledge the social relations embedded within traditional variables such as race/ethnicity, class, and gender. One way of doing that may involve a closer integration of positivist and critical realist investigations of health inequities [82,83].

### Socioeconomic Factors and Health Transitions

While many of the studies controlled for socioeconomic status as part of a statistical model, few focused on socioeconomic status as a central variable or reported its findings directly. Many simply indicated that the statistics presented where adjusted for income and/or education [see, for example: [39,44,45,52,73,84]. No study in this review used a class-based theoretical frame, and no study sought to theorize about structural factors that may drive health inequities, a limitation that is congruent with critiques of the dominance of positivism over critical realism in research on the social determinants of health [7,82]. When the effect of socioeconomic status was presented, these tended to follow expected gradient- or gradient-like patterns [19,20,54,56].

However, several important studies have presented results contrary to what would be expected from the standard social gradient model. As noted above, Smith et al's [61] analysis of the interaction between immigration, income, and gender as predictors of depression detected an intriguing effect, wherein male low-income recent immigrants reported lower levels of depression than male mid-/high-income recent immigrants. In contrast, female low-income recent immigrants fare poorly compared to middle- and high-income recent immigrants. In other words, a differential income effect for male and female recent immigrants may exist, with higher income being associated with an increased odds of depression for new male immigrants. Smith et al's interpretation of the pattern is that low-income new male immigrants may experience an advantage, "an absence of risk", and/or that low income represents a transitional state for male immigrants (with income not reflecting their wealth or status). More research is needed on this issue, as well as on a contrasting explanation that would see not an advantage for low-income immigrants but a disadvantage for mid-/high-income immigrants, who may - despite their high income - be exposed to different levels of workplace discrimination. This alternative explanation would be congruent with the findings of Auger et al [70] and Moore et al [71] discussed above, with higher socioeconomic status being associated with unexpectedly higher than expected health burdens.

Research on the health transitions of immigrants is perhaps uniquely positioned to contribute to the literature on the social patterning of health, insofar as it may disentangle the often-confounded effects of income, wealth, and status. However, for this to be accomplished, future studies will need to pay more attention to issues of inequality and poverty, and these - along with class - will need to become more central in research designs.

### Distinguishing Compositional and Contextual Effects

The majority of studies in this review have been based on individuals as the sole unit of analysis. A consequence of this is that the 'explanatory sphere' has been limited to compositional factors - characteristics of immigrants and Canadian-born respondents. This is an important limitation; one that could be overcome with the use of more advanced techniques based on multilevel modelling [13,59,85]. Such work could incorporate contextual factors as possible explanations for health transitions. For example, are health transitions of immigrants influenced by the level of income inequality in their community? Or are they influenced by the visibility/acceptance of their culture and religion in their area?

Only a few studies in this area have used multilevel techniques [58,70,72], and of these not all used multilevel analysis to explicitly model contextual effects. Stafford et al [58] do so, and their multilevel analyses indicate an intriguing pattern: as the percentage of immigrants in a region increased, the likelihood of depression for immigrants/visible minorities decreased but it increased for whites. There is therefore something about the place that matters to the health of both immigrants and the native-born. However, much more remains to be done in this area. Future studies could incorporate multilevel analysis and thereby integrate into the substantial literatures on social capital [86-88] and income inequality [89,90]. A multilevel approach could incorporate macro-level determinants (e.g., public policy at the level of the state, province, or city), as well as community-level factors (e.g., income distribution, social capital), and individual/compositional characteristics. The result could be a complex, nuanced, and useful account of the social determinants of health as experienced by different groups in different places.

## Conclusion

Empirical studies of the health of immigrants to Canada overwhelmingly support the notion that immigrants are healthier than the native-born population at the time of their arrival in the country but that this advantage may be lost over a relatively short period of time (2 to 10 years). This patterning is found, with a few exceptions, in studies of self-assessed health status and the presence of a diagnosis for chronic conditions such as diabetes and heart disease. Studies of overweight and obesity also support this notion, and introduce complexity by detecting heterogeneity in immigrant health status and trajectories. Most recently, researchers have begun to examine mental health and birth outcomes in relation to immigration to Canada. The emerging pattern appears to be that depression and other mental health issues are less prevalent among immigrants than the Canadian-born population. However, this advantage diminishes as length of residence in Canada increases. Living in areas with a high density of immigrants may help immigrants to retain this advantage. At the same time, immigrants who experience discrimination may be more likely to report worsening health. Additionally, income may interact with gender as a determinant of mental health, with mid- and high-income immigrants faring worse than expected given their income status.

The studies analysed in this review focused on chronic disease. Further research is needed on patterns with infectious disease, including tuberculosis and HIV/AIDS. Hyman, in a review of the evidence on the health of Canadian immigrants, notes that Canadian expert opinion is that the majority of tuberculosis cases among immigrants and refugees stem from previous infections that are re-activated post-migration, and that only a small subset of cases are the result of post-migration primary infection [5]. This would suggest that the health transitions of immigrants may be distinct when it comes to infectious diseases. However, using historical examples, Beiser [4] questions whether immigrants and refugees bring tuberculosis with them, or if they experience an increased risk of exposure as a result of overcrowding and poor living conditions. This may involve reactivation of latent tuberculosis or the development of a new infection. In the case of HIV/AIDS, little is known about the interaction of exposure to risk, acculturation, and immigration to countries such as Canada.

Research on the health transitions of immigrants to Canada has the potential to develop in theoretically and methodologically nuanced ways, and thereby contribute to wider debates on the social determinants of health and health inequities. Increasingly, researchers in this area are engaging with literature on identities, and may move from static classifications of 'race/ethnicity' to relational classifications of 'racialized identities' which sees categorizations such as 'visible minority' and 'non-white' as expressions of power. Integrating concepts like this into the statistical modelling that forms the heart of this research programme is a formidable challenge - but if successfully accomplished, would hold important lessons for medical sociology and social epidemiology in general.

At the same time, there are signs that the statistical models used in this area are advancing from ignoring structure to modelling it, thereby seeing health as a product not just of compositional factors such as age, gender, and income, but also of contextual factors - the characteristics of the places where people live and work. Along with these theoretical and methodological opportunities, research on the health transitions of immigrants to Canada may contribute to a more useful global social science through comparative analyses with other countries with high rates of immigration, including Australia [91] and the United States [1,92,93].

This review has focused exclusively on quantitative analyses of immigrant health. Most of the studies in the review relied on data from major Statistics Canada surveys, including the NPHS, the CCHS, and most recently, the LSIC. These surveys, whilst offering important properties, including national representativeness, do limit the kinds of questions that researchers investigate; work that values an interpretivist epistemology is simply not possible using these datasets [7]. Alternatively, qualitative methodologies may be used to better understand the challenges faced by service providers in seeking to provide care for immigrant groups [94], and participatory action research may be used to evaluate prevention and treatment programs [95]. However, researchers interested in determining the magnitude of health status differences between immigrants and the Canadian-born population will likely continue to rely on data sources such as the CCHS and the LSIC for the foreseeable future.

There are several limitations to this review. The first, as noted above, is an exclusive focus on quantitative studies. A second major limitation is a reliance on English-language publications. Future work should examine French-language studies. The review has also focused on adult health outcomes (and pregnancy-related outcomes such as low birth weight). Future work should extend the analysis to include child and adolescent health outcomes. Future work could also compare findings from Canada to other immigrant-receiving countries. Lastly, whilst the search strategy was designed to locate relevant studies, it is possible that not all studies have been identified. However, there is no reason to believe that studies have been excluded in a systematic way.

This is a rapidly growing area of research. More than a dozen studies have been published in the last 18 months, with more likely to come as a result of the release of data from the LSIC. A better understanding of the drivers of immigrant health is of utmost importance, particularly in a country like Canada, which has a long-standing tradition of research on the social determinants of health [96].

## Competing interests

The author declares that they have no competing interests.
